# Heteroglycoclusters With Dual Nanomolar Affinities for the Lectins LecA and LecB From *Pseudomonas aeruginosa*

**DOI:** 10.3389/fchem.2019.00666

**Published:** 2019-10-02

**Authors:** David Goyard, Baptiste Thomas, Emilie Gillon, Anne Imberty, Olivier Renaudet

**Affiliations:** ^1^Univ. Grenoble Alpes, CNRS, DCM UMR 5250, Grenoble, France; ^2^Univ. Grenoble Alpes, CNRS, CERMAV, Grenoble, France

**Keywords:** heteroglycocluster, multivalency, orthogonal ligation, lectin, *Pseudomonas aeruginosa*

## Abstract

Multivalent structures displaying different instead of similar sugar units, namely heteroglycoclusters (hGCs), are stimulating the efforts of glycochemists for developing compounds with new biological properties. Here we report a four-step strategy to synthesize hexadecavalent hGCs displaying eight copies of αFuc and βGal. These compounds were tested for the binding to lectins LecA and LecB from *Pseudomonas aeruginosa*. While parent fucosylated (**19**) and galactosylated (**20**) homoclusters present nanomolar affinity with LecB and LecA, respectively, we observed that hGCs combining these sugars (**11** and **13**) maintain their binding potency with both lectins despite the presence of an unspecific sugar. The added multivalency is therefore not a barrier for efficient recognition by bacterial receptors and it opens the route for adding different sugars that can be selected for their immunomodulatory properties.

## Introduction

Glycoclusters and glycodendrimers remain a growing source of interest in glycosciences (Renaudet and Roy, 2013). For a long time, synthetic chemists focused their efforts on structures displaying multiple copies of a single sugar unit to both clarify and modulate biological and pathological processes involving multivalent interactions with proteins (Bernardi et al., 2013; Cecioni et al., 2015; Arsiwala et al., 2019). However, these compounds only partially reflect the heterogeneous expression of glycans at the cell surface (i.e., glycocalyx) and completely underestimate that other sugar units may participate or promote additional biological events (Jiménez Blanco et al., 2013; Müller et al., 2016). For this reason, the development of structures displaying different rather than unique sugars, namely heteroglycoclusters (hGCs), as mimics of the glycocalyx is renewing the enthusiasm in this field.

So far, several heterovalent structures have been reported for studying the interaction process with a large class of carbohydrate-specific proteins (i.e., glycosidases, glycosyltransferases, lectins, antibodies) (Deguise et al., 2007; Ortega-Muñoz et al., 2009; Gómez-García et al., 2010; Lindhorst et al., 2010; Karskela et al., 2012; Abellán Flos et al., 2016; Vincent et al., 2016; Ortiz Mellet et al., 2017; Gade et al., 2018; Ogura et al., 2018) or for triggering multifaceted immune response against tumor cells (Ragupathi et al., 2002; Zhu et al., 2009; Pett et al., 2017). These studies highlight the complexity of heterocluster effects and reinforce the need of new structures to go one step further toward the understanding of naturally occurring recognition events and the development of more active compounds. Among recent reports, J. M. Garcia Fernandez and coworkers have demonstrated the impact of heterovalent display in both glycosidase inhibition and lectin binding using a competitive enzyme-lectin binding assay (Abellán Flos et al., 2016; García-Moreno et al., 2017). In addition, the Wong group have shown by glycan array that ligand density and neighboring sugars significantly affect the binding with antibodies (Liang et al., 2011). In another study, Reymond and co-workers focused their interest on the lectins LecA and LecB from *Pseudomonas aeruginosa* (Michaud et al., 2016). These two lectins play central roles in both the adhesion of the bacteria to host cells and the biofilm formation, causing severe damages in particular for immunocompromised patients (Mitchell et al., 2002; Imberty et al., 2004). Instead of using homovalent structures specific for either LecA or LecB, the authors postulated that the utilization of compounds combining both α-fucose (αFuc) and β-galactose (βGal) could bind simultaneously LecA and LecB, thus improving inhibitory effect to fight this antibiotic resistant bacterium. To this aim, glycodendrimers decorated with βGal and αFuc have been synthesized and promising activity in comparison with their previous homoclusters has been observed. This pioneering study prompted us to design new heterovalent glycodendrimers and to study their ability to bind these two lectins with high affinity and without loss of efficiency.

In the course of developing multivalent glycoconjugates (Galan et al., 2013; Daskhan et al., 2015), we recently focused on the synthesis of a variety of cyclopeptide-based hGCs using orthogonal chemoselective conjugation methods such as the oxime ligation (OL), the Cu(I)-catalyzed azide-alkyne cycloaddition (CuAAC), and the thiol-ene, thiol-chloroactetyl and diethyl squarate couplings (TEC, TCC, DSC). We thus synthesized diverse 4-, 8-, and 16-valent structures displaying from 2 to 4 different sugars in well-defined shuffled proportion and disposition (Thomas et al., 2012; Daskhan et al., 2016; Pifferi et al., 2017). In addition, sequential one-pot multi-click and iterative divergent strategies gave access to glycoconjugates with unprecedented structural complexity in excellent yields and purity (Thomas et al., 2015a). In the present study, we capitalized on these methodologies to synthesize glycodendrimers bearing βGal and αFuc with different chemical linkage and evaluated their binding to LecA and LecB. Because αMan is ligand of LecB, though with a lower affinity than αFuc, we also synthesized different combinations of hGCs containing this residue to evaluate its potential influence in the binding to these two lectins.

## Results and Discussion

**Figure 1:**
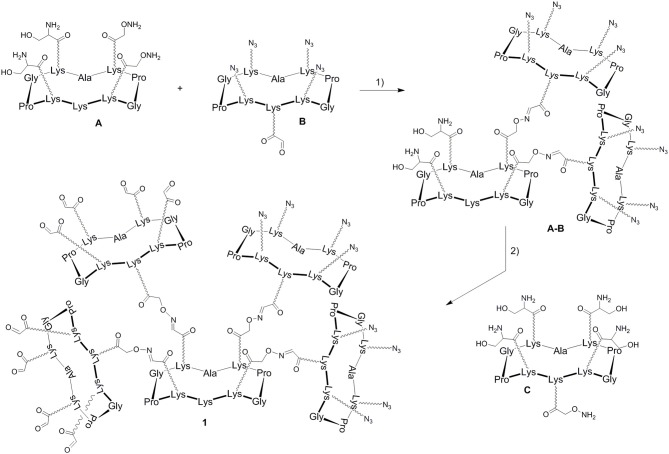
Strategy for the construction of the hexadecavalent scaffold 1. Reagents and conditions (Pifferi et al., 2017): (1) 0.1% TFA in H_2_O/CH_3_CN (1:1), 37°C, 30 min; (2) i: NaIO_4_, H_2_O, r.t., 40 min; ii: 0.1% TFA in H_2_O/CH_3_CN (1:1), 37°C, 30 min; (3) NaIO_4_, H_2_O, r.t., 40 min.

**Figure 2:**
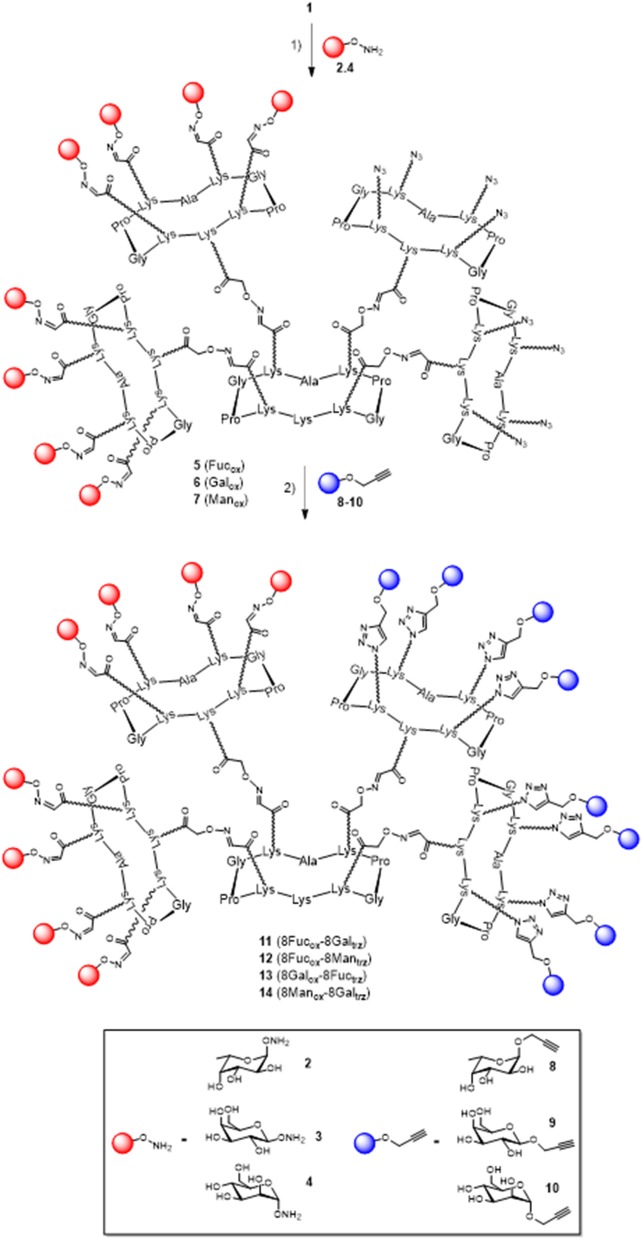
Synthesis of hGCs **11**–**14** by a mixed OL and CuAAC strategy. Reagents and conditions: (1) **2**, **3**, or **4**, 0.1% TFA in H_2_O/CH_3_CN, 37°C, 30 min; (2) **8**, **9**, or **10**, CuSO_4_, Na ascorbate, THPTA, PBS (pH 7.4, 10 mM), r.t., 90 min, 81% for **11**, 84% for **12**, 77% for **13**, and 76% for **14**.

In a previous report, we described a series of homovalent glycodendrimers with nanomolar affinity for LecB (Berthet et al., 2013). These compounds have been prepared by a convergent oxime conjugation of cyclopeptides and/or polylysine dendrons then aminooxylated sugar units have been grafted at the periphery. However, the same strategy is not compatible to introduce two different sugars in a regioselective and controlled manner (Bossu et al., 2011). Instead, we used here OL and CuAAC to secure the molecular assembly and the final purification of complex structures (Thomas et al., 2015b). To do so, the construction of the dendrimer core was first carried out from a central cyclopeptide **A** (Figure 1) containing two aminooxy groups and two serine as oxo-aldehyde precursors as previously described (Pifferi et al., 2017). This scaffold **A** was successively functionalized with two cyclopeptides: the first one **B** (right arm) contains one aldehyde and four azido groups, whereas the second one **C** (left arm) displays one aminooxy and four serines. Treatment with sodium periodate of the resulting hexadecavalent dendrimer afforded **1** which was functionalized with aminooxylated αFuc (**2**), βGal (**3**), and αMan (**4**) in aqueous solution containing 0.1% TFA at 37°C for 30 min. These three octavalent conjugates displaying eight copies of αFuc (**5**), βGal (**6**), and αMan (**7**) were subsequently conjugated without further purification with propargylated αFuc (**8**), βGal (**9**), and/or αMan (**10**) in PBS (pH 7.4, 10 mM) with CuSO_4_, tris(3-hydroxypropyltriazolylmethylamine) (THPTA) and sodium ascorbate (Figure 2). After RP-HPLC purification, the resulting hGCs **11**–**14** were obtained in 76–84% yield.

**Figure 3:**
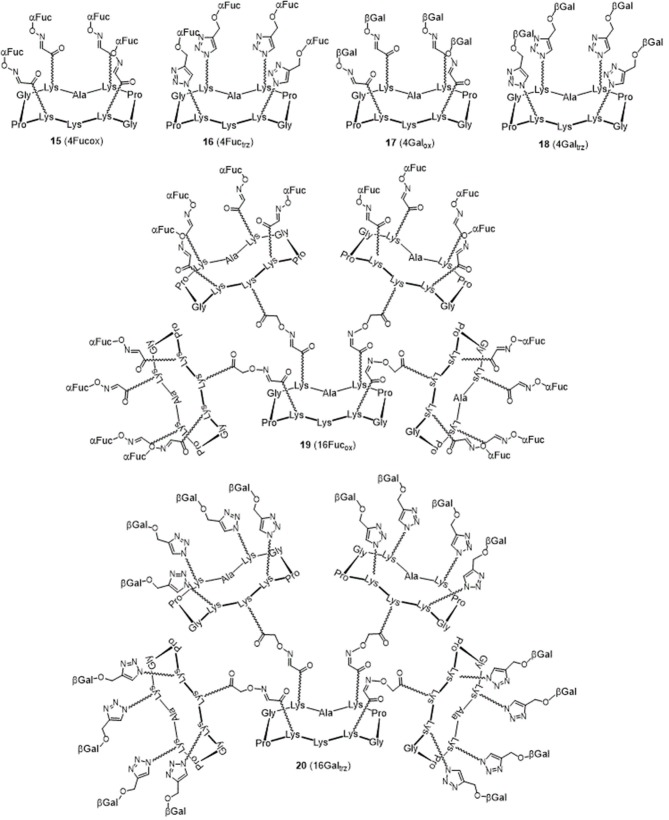
Structure of the tetravalent (**15–18**) and hexadecavalent (**19–20**) homoclusters.

The resulting hGCs were tested in solution with lectins by Isothermal Titration Calorimetry (Table 1) and their binding properties were compared with homoclusters **15**–**20** (Berthet et al., 2013; Thomas et al., 2015b) displaying either 4 and 16 βGal or αFuc units attached with an oxime or a triazole linker (Figure 3). Methyl α-L-fucoside (αFuc-OMe) and methyl β-D-galactoside (βGal-OMe) were used as monovalent references for LecB and LecA to calculate the relative potency (α) and relative potency per sugar (β) of each multivalent compound. Experiments were performed in direct injection mode (i.e., ligand in syringe and protein in cell) to minimize aggregation problems.

**Table 1:** Isothermal titration microcalorimetry data for binding to LecA and LecB^a^.

**Lectin**	**Compound**	**Kd [nM]**	**n**	**–ΔH [kJ.mol^**−1**^]**	**–ΔG [kJ.mol^**−1**^]**	**−TΔS [kJ.mol^**−1**^]**	**α**	**β**
LecB	αFuc-OMe^b^	430	0.77	41	36.4	−5	1	1
	**15** (4Fuc_ox_)	334 ± 35	0.17	103.5 ± 2	37.1	66.5	1.3	0.3
	**16** (4Fuc_trz_)	367 ± 26	0.19	119.5 ± 1	36.8	82.8	1.2	0.3
	**19** (16Fuc_ox_)	44 ± 5	0.06	321.0 ± 14	42.0	279.0	9.8	0.6
	**11** (8Fuc_ox_-8Gal_trz_)	92 ± 14	0.10	212.1 ± 6	40.2	171.9	4.7	0.8
	**12** (8Fuc_ox_-8Man_trz_)	85 ± 16	0.08	211.0 ± 3	40.4	170.6	5.1	0.6
	**13** (8Gal_ox_-8Fuc_trz_)	118 ± 15	0.10	182.9 ± 8	39.5	143.3	3.6	0.5
LecA	βGal-OMe^c^	150,000	0.8	39	22	15	1	1
	**17** (4Gal_ox_)	91 ± 4	0.32	139.5 ± 5	40.2	99.6	1,648	412
	**18** (4Gal_trz_)	22 ± 2	0.26	139.0 ± 1	43.8	95.5	6,818	1,705
	**20** (16Gal_trz_)	14 ± 0.7	0.09	378.6 ± 15	44.8	333.8	10,714	670
	**11** (8Fuc_ox_-8Gal_trz_)	34 ± 7	0.16	232.6 ± 2	42.6	189.9	4,412	551
	**13** (8Gal_ox_-8Fuc_trz_)	35 ± 0.1	0.16	216 ± 3	42.5	173.6	4,286	536
	**14** (8Man_ox_-8Gal_trz_)	21 ± 4	0.16	243.2 ± 19	43.8	199	7,142	893

a *Thermodynamic data are referred to moles of glycoclusters and stoichiometry expressed as the number of glycocluster molecules per lectin monomer. Standard deviations are indicated on experimentally derived values (at least two experiments). α factor is the relative potency compared to the monovalent compound. β factor is the relative potency per sugar unit*.

b *see Berthet et al. (2013)*.

c *see Cecioni et al. (2011)*.

**Figure 4:**
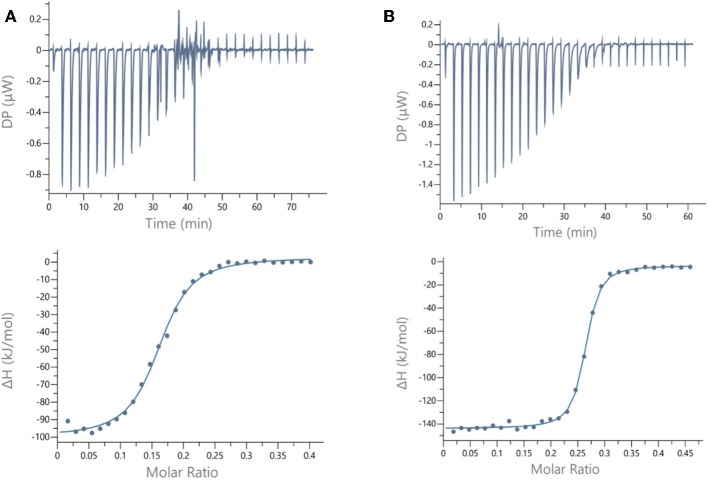
ITC data. Thermograms obtained by injections of **(A)** glycocluster **16** at 140 μM in a solution of LecB (70 μM) and **(B)** glycocluster **18** at 80 μM in a solution of LecA (35 μM) with corresponding integrated titration curves. Molar ratio is indicated as number of glycocluster molecules per lectin monomer.

For LecB, we observed modest and similar *K*d (0.3 μM) for tetravalent compounds **15** and **16**, with negligible improvement factor α compared to αFuc-OMe indicating the absence of cluster effect (Table 1, Figure 4A, and Supplementary Information). Instead strong aggregation is observed as shown on the thermogram in Figure 4A. The analysis of the thermodynamic contributions indicates a strong gain in enthalpy but that is completely counterbalanced by the entropy cost. This again indicates that the tetravalent compound is bridging between different lectin tetramers instead of clustering neighboring binding sites.

By contrast, for LecA, a very strong improvement of binding was observed for both tetravalent compounds **17** and **18** (*K*d = 22 nM) compared to βGal-OMe (Table 1). This suggests a favorable presentation of sugar for the binding to LecA (Cecioni et al., 2012) with geometry allowing for a real clustering effect, also reflected in the huge enthalpy gain. Compound **18** that contains a triazole linkage instead of oxime (**17**) is the most efficient with a factor α of 7,000 and factor β of 1,700 when reported to the number of sugar (Table 1, Figure 4B, and Supplementary Information). This result is in good agreement with the well-known preference of LecA for β-galactoside containing an aromatic moiety near the anomeric position (Cecioni et al., 2012).

Interestingly, multimerisation of the tetravalent structures **15** and **18** as the hexadecavalent dendrimers **19** and **20** allowed 10-fold binding improvement for fucosylated compounds with LecB (*K*d = 44 nM for **19**). Only minor difference in *K*d was observed between **18** and **20** and LecA (*K*d = 22 and 14 nM, respectively), despite a more favorable binding enthalpy for **20** (–ΔH = 378 kJ/mol) and a higher improvement factor α (10,000) compared to βGal-OMe. While both **18** and **20** are excellent ligands for LecA, compound **20** shows a 2.5-lower β factor than **18** which suggests that increasing valency over four sugars units has low effect for enhancing affinity with LecA. Similar observations have been already reported in other studies (Cecioni et al., 2012; Michaud et al., 2016).

**Figure 5:**
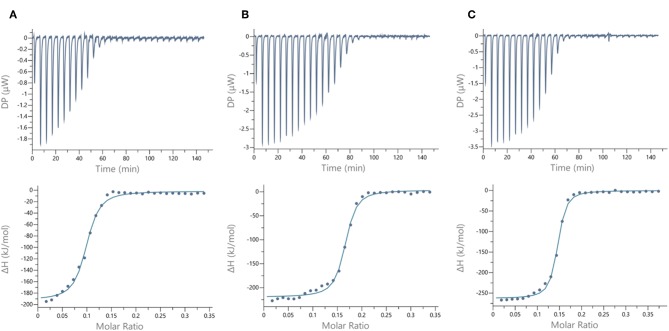
ITC data. Thermograms obtained by injections of **(A)** glycodendrimer **11** at 50 μM in a solution of LecB (32 μM), **(B)** glycodendrimer **11** (50 μM) in a solution of LecA (32 μM), and **(C)** glycodendrimer **14** (40 μM) in a solution of LecA (29 μM) with corresponding integrated titration curves. Molar ratio is indicated as number of glycocluster molecules per lectin monomer.

We next evaluated the binding potency of hGCs for LecA and LecB by ITC (Table 1, Figure 5, and Supplementary Information). Two compounds **11** and **13** display eight copies of αFuc and βGal with either oxime or triazole linkers and two other hGCs combine αMan with αFuc (**12**) or βGal (**14**). As observed with tetravalent clusters **15**–**16** with LecB, a higher affinity (*K*d = 85–92 nM) was measured when the fucose unit is linked by an oxime linkage. These compounds **11** and **12** showed a more favorable binding enthalpy (–ΔH = 212 kJ/mol), suggesting a more favorable geometry and presentation of fucose. Moreover, no significant difference of affinity was observed between **11** and **12** with binding improvement in the same range of magnitude than homovalent compounds (i.e., β factor lower than 1), indicating the absence of influence of the partner sugar (Man or Gal).

As for LecA, as mentioned with homovalent structures **17**–**20**, hGCs displaying βGal **11**, **13**, and **14** lead to similar *K*d (21–35 nM), showing strong binding entropy contribution (–ΔS = 173–199 kJ/mol) counterbalanced by favorable binding enthalpy (–ΔH = 216–243 kJ/mol).

When the binding is compared to parent galactosyl-homoclusters, the β factor was ~500 for both βGal-αFuc hGCs **11** and **13** and 900 for **14** which displays βGal-αMan combination. The stoichiometry data for these three compounds indicated that approximately six βGal can bind to a monomer of LecA, which indicates a good accessibility despite the presence of non-specific sugars close to βGal. This results supports the fact that high affinity could be due to an aggregative binding mode. In the case of LecB, the stoichiometry measured for **11**–**13** (*n* = 0.1) lead to a similar hypothesis. Altogether, our data suggest that hGCs combining both βGal and αFuc derivatives not only bind to both lectins with nanomolar affinity, but also that the presence of the other sugar does not affect the binding potency.

## Conclusion

We have developed a synthetic strategy to prepare a series of heteroglycoclusters displaying eight copies of αFuc and βGal. Binding assays with both LecA and LecB lectins from *Pseudomonas aeruginosa* have revealed that the combination of αFuc and βGal with unspecific sugars maintain the binding potency of the parent homoclusters with both lectins. The review by Jiménez Blanco et al. (2013) opened the questions of the effect of functional promiscuity of glycoligands on different heteroglycocompounds, since such complex architecture are efficient mimics of cell surfaces. The authors raised concerns that the complexity may create “messiness or noise in the processes they participate in.” We could demonstrate here that the crowding with an alternate sugar does not lower the efficiency of the glycocluster to bind to the bacteria lectins. In the future, this could be of importance for designing active compounds that will have the capacity of both binding to the bacterial surface and activating the immune system by different clusters of sugars.

## Experimental Section

### Materials

All chemical reagents were purchased from Aldrich (Saint Quentin Fallavier, France) or Acros (Noisy-Le-Grand, France). All protected amino acids and Fmoc-Gly-Sasrin® resin was obtained from Advanced ChemTech Europe (Brussels, Belgium). For peptides and glycopeptides, analytical RP-HPLC was performed on Waters system equipped with a Waters 2695 separations module and a Waters 2487 Dual Absorbance UV/Visible Detector. Analysis was carried out at 1.0 mL/min (EC 125/3 nucleosil 300-5 C_18_) with UV monitoring at 214 nm and 250 nm using a linear A–B gradient (buffer A: 0.09% CF_3_CO_2_H in water; buffer B: 0.09% CF_3_CO_2_H in 90% acetonitrile). Purifications were carried out at 22.0 mL/min (VP 250/21 nucleosil 100-7 C_18_) with UV monitoring at 214 nm and 250 nm using a linear A–B gradient. HRMS and ESI-MS and HRMS spectra of peptides and glycopeptides were measured on an Esquire 3000 spectrometer from Bruker. Lectins LecA and LecB were produced in recombinant form in *Escherichia coli* BL21(DE3) as described previously (Mitchell et al., 2005; Blanchard et al., 2008).

### Synthetic Procedures

#### Synthesis of Compound 11

Cyclopeptide **1** (4.5 mg, 0.72 μmol) and aminooxy Fuc **2** (1.6 mg, 8.7 μmol) were dissolved in 0.1% TFA in H_2_O (10 mM). After stirring for 30 min at room temperature, the propargyl Gal **9** (5.1 mg, 23.2 μmol) dissolved in DMF (1 mL) and a solution of CuSO_4_ (1,4 mg, 5.8 μmol) in PBS buffer (500 μL, 100 mM) were added. Then was added a solution of THPTA (10.1 mg, 23.2 μmol) and sodium ascorbate (5.1 mg, 40.1 μmol) in PBS buffer (500 μL, 100 mM). All solutions were previously degassed under argon. The reaction was stirred at room temperature under argon and after 1 h analytical HPLC indicated complete reaction coupling. Then Chelex resin was added to remove excess of copper and the reaction mixture was directly purified by RP-HPLC affording pure compound as a white powder. Yield: 81% (5.4 mg, 0.59 μmol); RP-HPLC: Rt = 4.28 min (C_18_, 214 nm 5–100% B in 15 min); MS (ESI^+^) *m/z* calcd. for C_387_H_619_N_103_O_158_ [M+5H]^5+^: 1847.3, found 1847.4.

#### Synthesis of Compound 12

Compound **12** was obtained from compound **1**, aminooxy Fuc **2**, and propargyl Man **10** following the procedure described for **11**. Yield: 84% (5.6 mg, 0.61 μmol); RP-HPLC: Rt = 4.33 min (C_18_, 214 nm 5–100% B in 15 min); MS (ESI^+^) *m/z* calcd. for C_387_H_618_N_103_O_158_ [M+4H]^4+^: 2309.1, found 2308.7

#### Synthesis of Compound 13

Compound **13** was obtained from compound **1**, aminooxy Gal **3** and propargyl Fuc **8** following the procedure described for **11**. Yield: 77% (3.5 mg, 0.38 μmol); RP-HPLC: Rt = 4.34 min (C_18_, 214 nm 5–100% B in 15 min); MS (ESI^+^) *m/z* calcd. for C_387_H_619_N_103_O_158_ [M+5H]^5+^: 1847.3, found 1847.3.

#### Synthesis of Compound 14

Compound **14** was obtained from compound **1**, aminooxy Man **4** and propargyl Gal **9** following the procedure described for **11**. Yield: 76% (3.1 mg, 0.33 μmol); RP-HPLC: Rt = 10.62 min (C_18_, 214 nm 5–100% B in 20 min); MS (ESI^+^) *m/z* calcd. for C_387_H_619_N_103_O_166_ [M+5H]^5+^: 1873.7, found: 1874.1.

#### Synthesis of Compound 15

Aldehyde-containing cyclopeptide (Singh et al., 2005) (10.0 mg, 8.04 μmol) and aminooxy Fuc **2** (8.6 mg, 48.23 μmol) were dissolved in 0.1% TFA in H_2_O (10 mM). After stirring for 30 min at room temperature, the crude mixture was purified by RP-HPLC. Fractions containing the product were combined and lyophilized to afford the title compound as a white fluffy powder (12.8 mg, 6.75 μmol, 84%). RP-HPLC: Rt = 4.77 min (C_18_, 214 nm 5–60% B in 15 min); HRMS (ESI^+^) *m/z* calcd. for C_79_H_130_N_19_O_34_ [M+H]^+^: 1888.9027, found: 1888.9058.

#### Synthesis of Compound 16

The title compound was prepared according to already published protocol. Analytical data were in agreement with the literature (Ribeiro et al., 2018).

#### Synthesis of Compound 17

The title compound was prepared following the procedure described for **15** using aminooxy Gal **3** (7.5 mg, 38.6 μmol) and was obtained as a white fluffy powder after lyophilisation (13.7 mg, 7.02 μmol, 80%). RP-HPLC: Rt = 3.38 min (C_18_, 214 nm 5–60% B in 15 min); HRMS (ESI^+^) *m/z* calcd. for C_79_H_130_N_19_O_38_ [M+H]^+^: 1952.8824, found: 1952.8890.

#### Synthesis of Compound 18

The title compound was prepared following the procedure described for **16** from propargyl Gal **9** (13 mg, 58.7 μmol) and was obtained as a white fluffy powder after lyophilisation (13.7 mg, 7.02 μmol, 80%). RP-HPLC: Rt = 4.17 min (C_18_, 214 nm 5–60% B in 15 min); HRMS (ESI^+^) *m/z* calcd. for C_83_H_134_N_22_O_34_ [M+H]^+^: 1996.9464, found: 1996.9428.

#### Synthesis of Compound 19

The title compound was prepared according to already published protocol. Analytical date were in agreement with the literature (Berthet et al., 2013).

#### Synthesis of Compound 20

The title compound was prepared according to the same protocol as compound **18** and was obtained as a white fluffy powder after lyophilisation (4.8 mg, 0.51 μmol, 69%). RP-HPLC: Rt = 3.98 min (C_18_, 214 nm 5–60% B in 15 min); MS (ESI^+^) *m/z* calcd. for C_395_H_628_N_111_O_158_ [M+7H]^7+^: 1350.2, found: 1351.8

### Isothermal Titration Microcalorimetry

ITC experiments of compounds **15–18** were perfomed with a PEAQ-ITC titration calorimeter (Microcal). Other compounds were assayed with a VP-ITC isothermal titration calorimeter (Microcal). The experiments were carried out at 25°C. All glycocompounds and lectins LecB and LecA were dissolved in the same buffer composed of 20 mM Tris with 100 mM NaCl and 0.1 mM CaCl_2_ at pH 7.5. For PEAQ-ITC, the lectins were placed in the microcalorimeter cell (200 μL) at concentrations varying from 35 to 70 μM. A total of 29 injections of 1.3 μL of glycoclusters at concentrations varying from 80 to 140 μM were performed. For VP-ITC, the microcalorimeter cell (1.447 mL) contained the lectins with concentrations between 30 and 100 μM. A total of 30 injections of 10 μL were performed intervals of 5 min while stirring at 310 rpm with glycoclusters concentrations varying from 0.40 to 0.150 μM. The experimental data were fitted to a theoretical titration curve using the Microcal PEAQ-ITC analysis software, with ΔH (enthalpy change), Ka (association constant), and N (number of binding sites per monomer) as adjustable parameters. Dissociation constant (Kd), free energy change (ΔG), and entropy contributions (TΔS) were derived from the previous ones. Two or three independent titrations were performed for each ligand tested.

## Data Availability Statement

All datasets generated for this study are included in the manuscript/Supplementary Files.

## Author Contributions

OR designed the study. DG and BT synthetized the compounds. DG and EG performed the ITC analysis. DG, AI, and OR wrote the manuscript.

### Conflict of Interest

The authors declare that the research was conducted in the absence of any commercial or financial relationships that could be construed as a potential conflict of interest.
