# Supplementary material for: Heteroglycoclusters With Dual Nanomolar Affinities for the Lectins LecA and LecB From Pseudomonas aeruginosa
Source: Front Chem. 2019 Oct 2;7:666. doi: 10.3389/fchem.2019.00666 (PMC6783499; doi:10.3389/fchem.2019.00666)
Supplement: Supplementary file 1 [file Data_Sheet_1.PDF]

# Table of contents

RP-HPLC and Mass Spectra ........................................................................... 2

Isothermal Titration Calorimetry: Titration curves and thermograms ........................ 12

References ........................................................................................................ 14

## RP-HPLC and Mass Spectra

## Compound 11

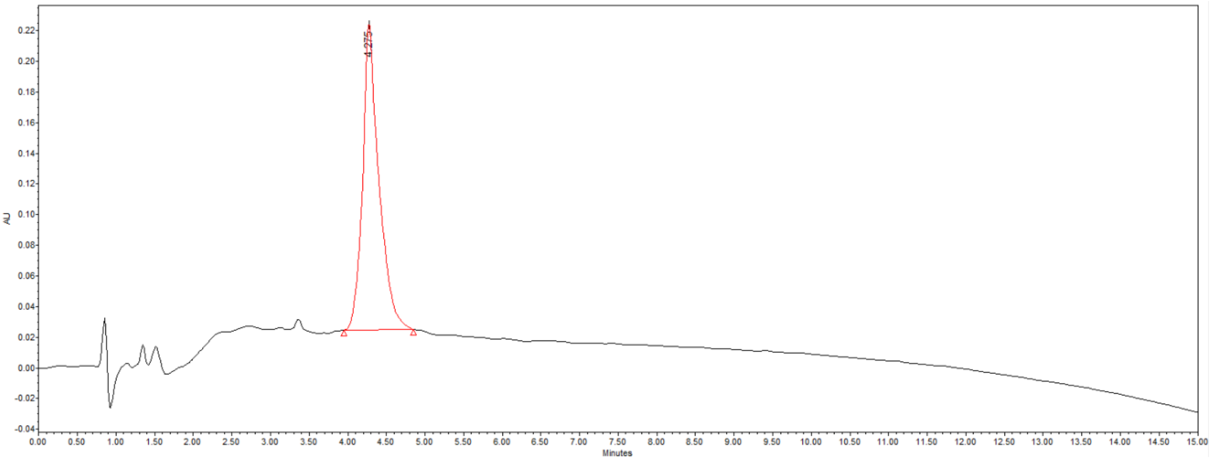


Figure S 1. RP-HPLC spectrum of compound **11**

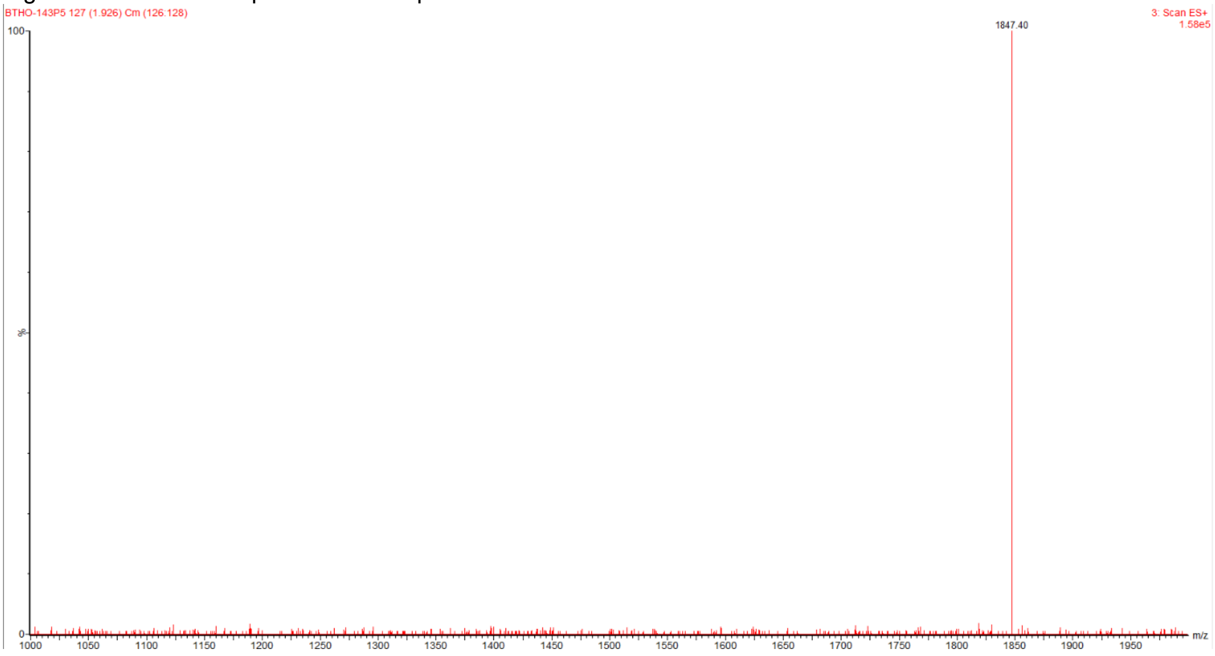


Figure S 2. ESI-MS spectrum of compound **11**

# Compound 12

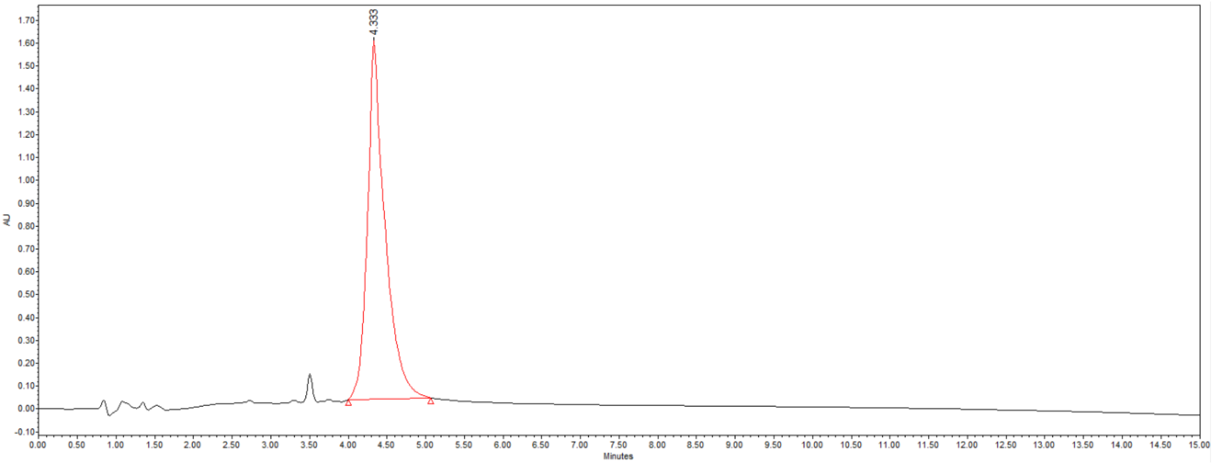


Figure S 3. RP-HPLC spectrum of compound **12**

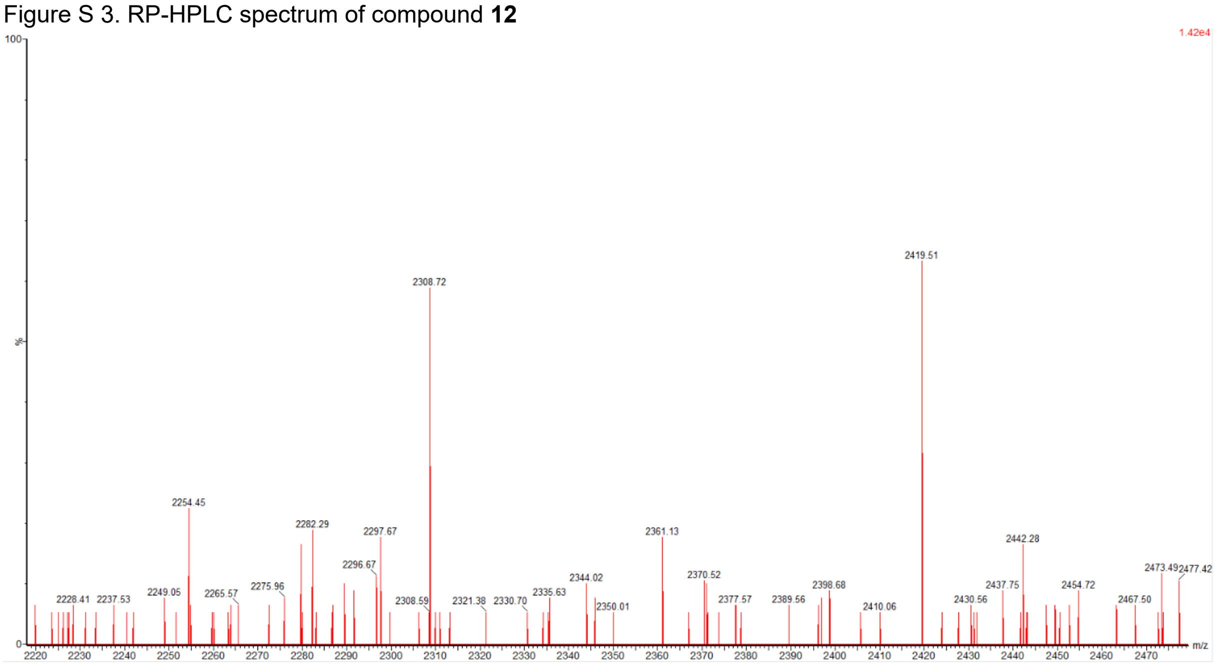


Figure S 4. ESI-MS spectrum of compound **12**

## Compound 13

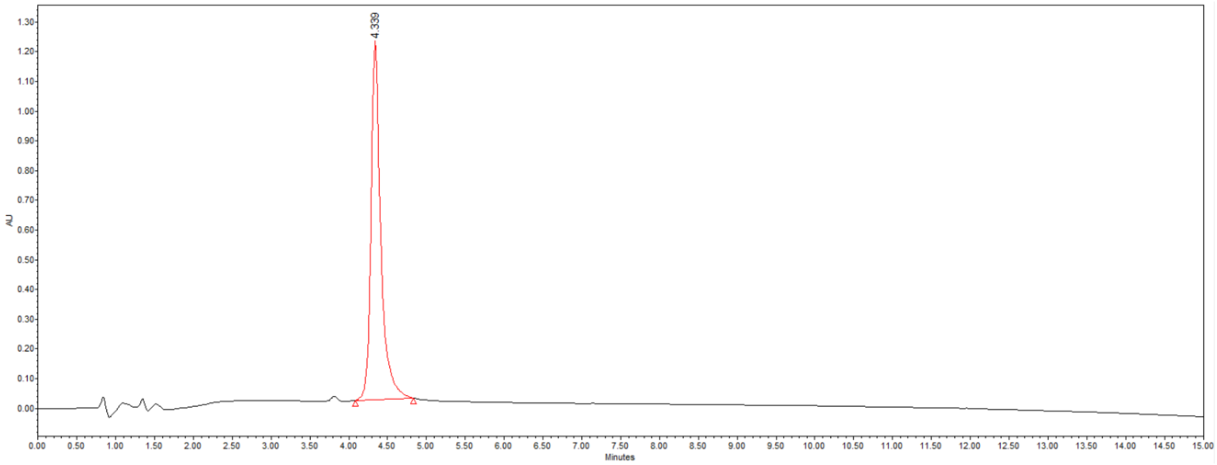


Figure S 5. RP-HPLC spectrum of compound **13**

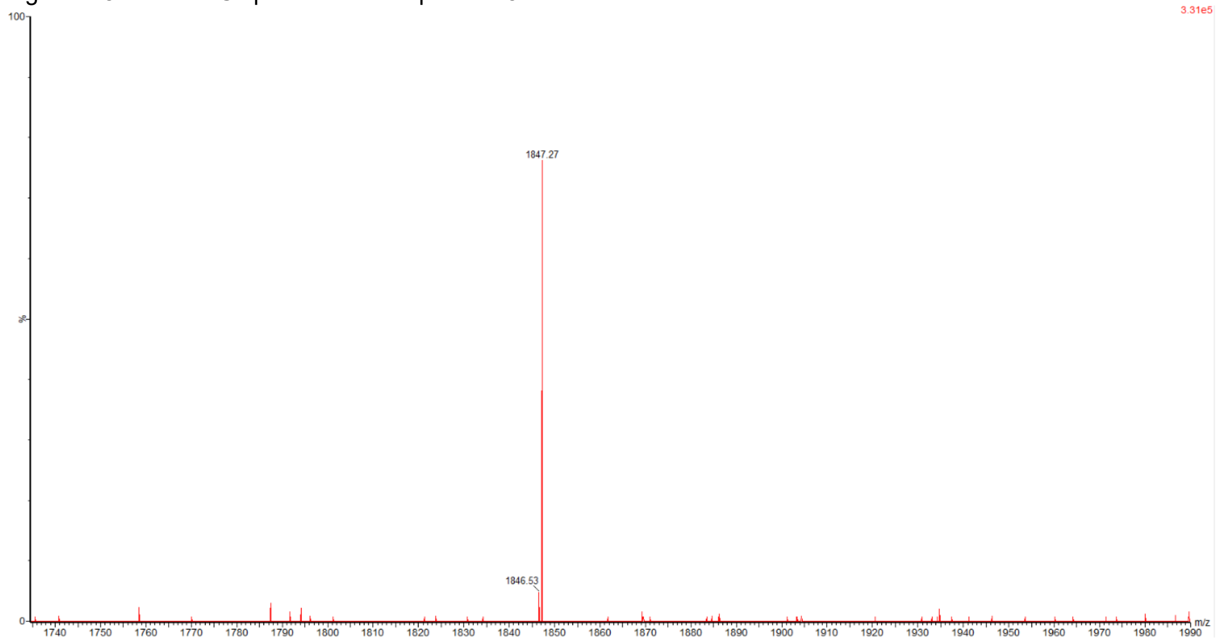


Figure S 6. ESI-MS spectrum of compound **13**

## Compound 14

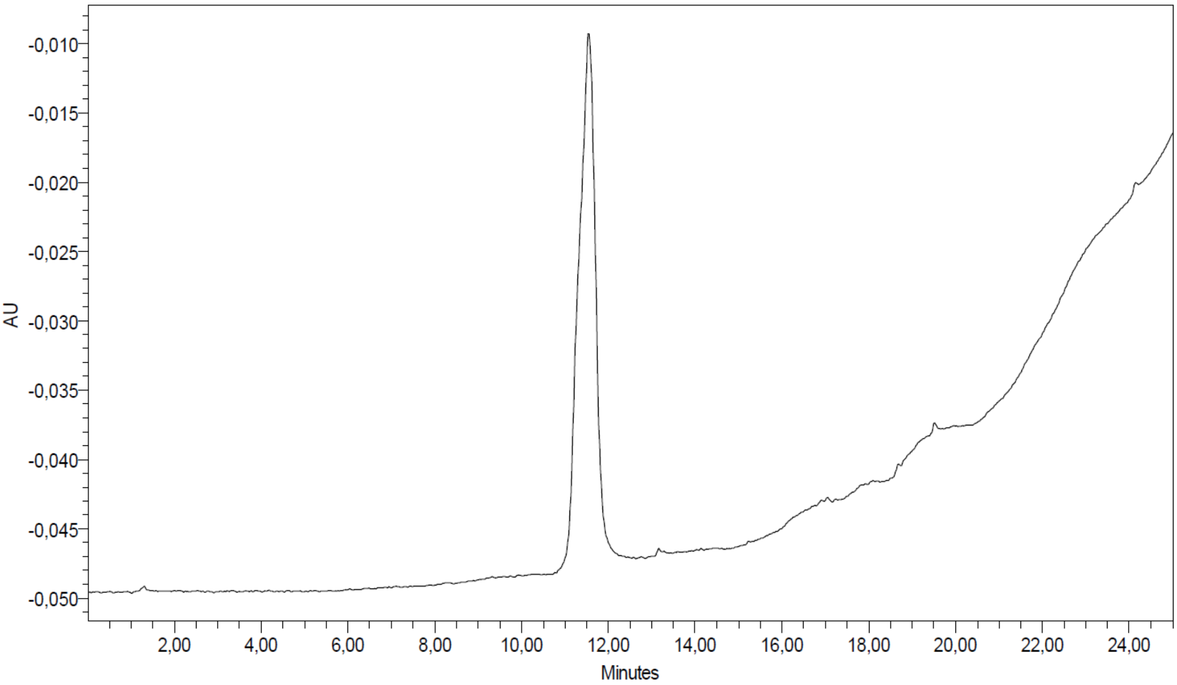


Figure S 7. RP-HPLC spectrum of compound **14**

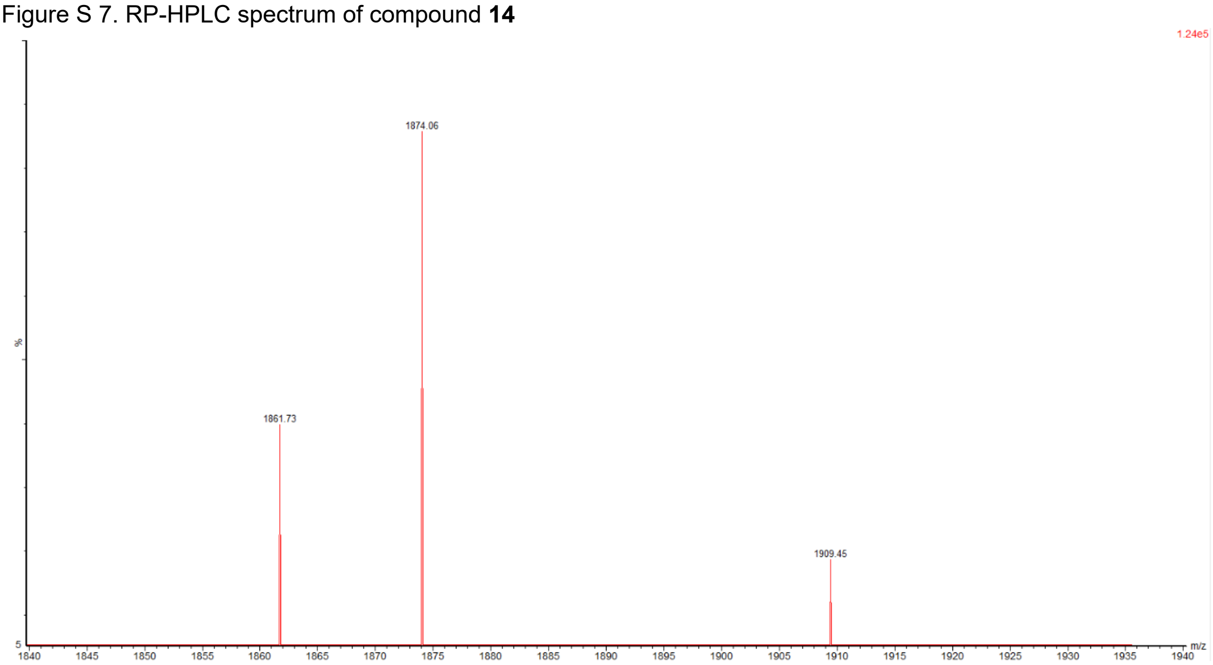


Figure S 8. ESI-MS spectrum of compound **14**

## Compound 15

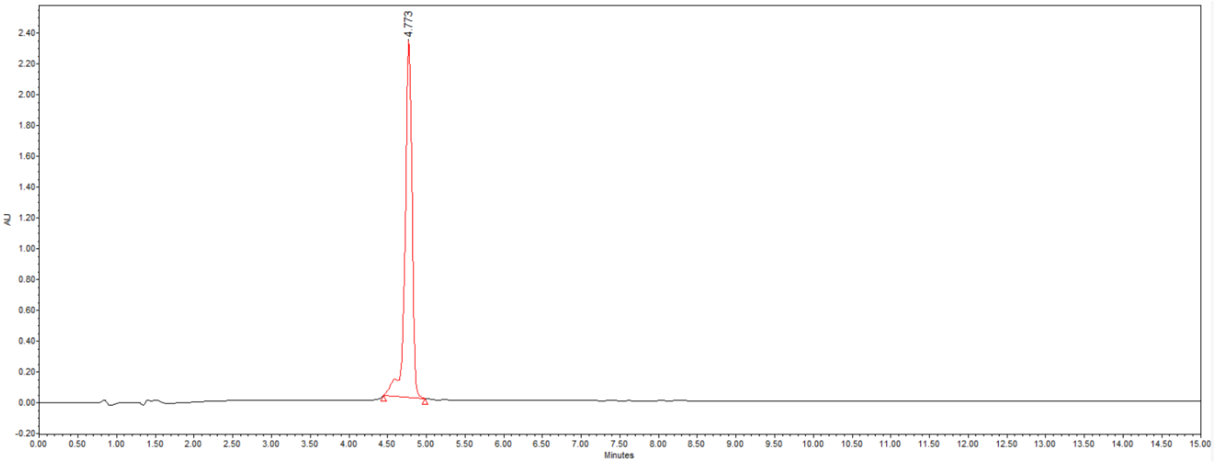


Figure S 9. RP-HPLC spectrum of compound **15**

| Target | Mass Found | Error PPM | Compound |
|---|---|---|---|
| 1888.9027 | 1888.90 | 1.6 | Found |

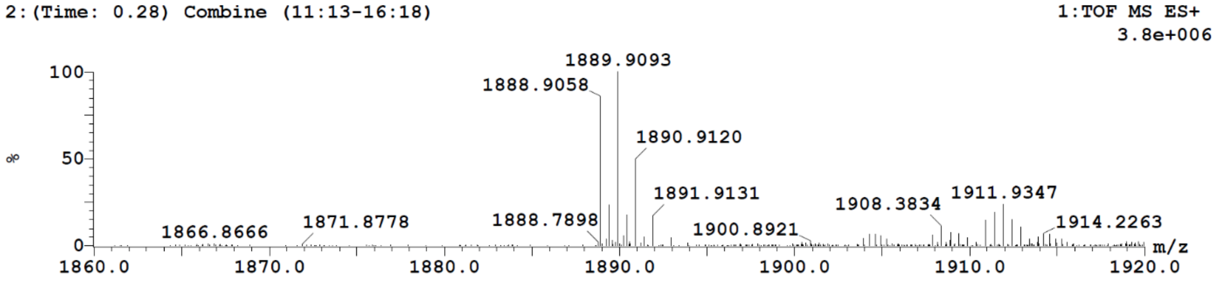


Figure S 10. HRMS spectrum of compound **15**

## Compound 16

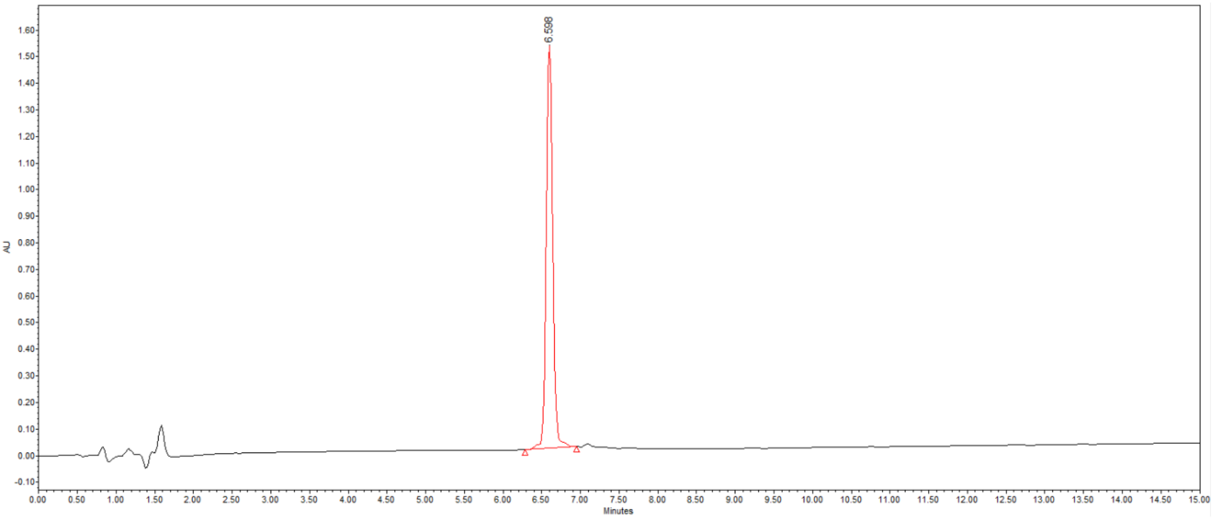


Figure S 11. RP-HPLC spectrum of compound **16**

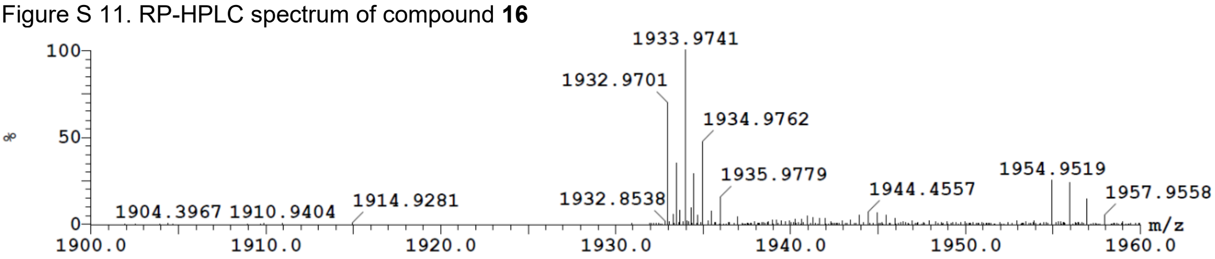


| Mass | PPM | i-FIT (norm) | Formula | Calc. Mass |
|---|---|---|---|---|
| 1932.9701 | 1.8 | 1.4 | C83 H134 N23 O30 | 1932.9667 |

Figure S 12. HRMS spectrum of compound **16**

# Compound 17

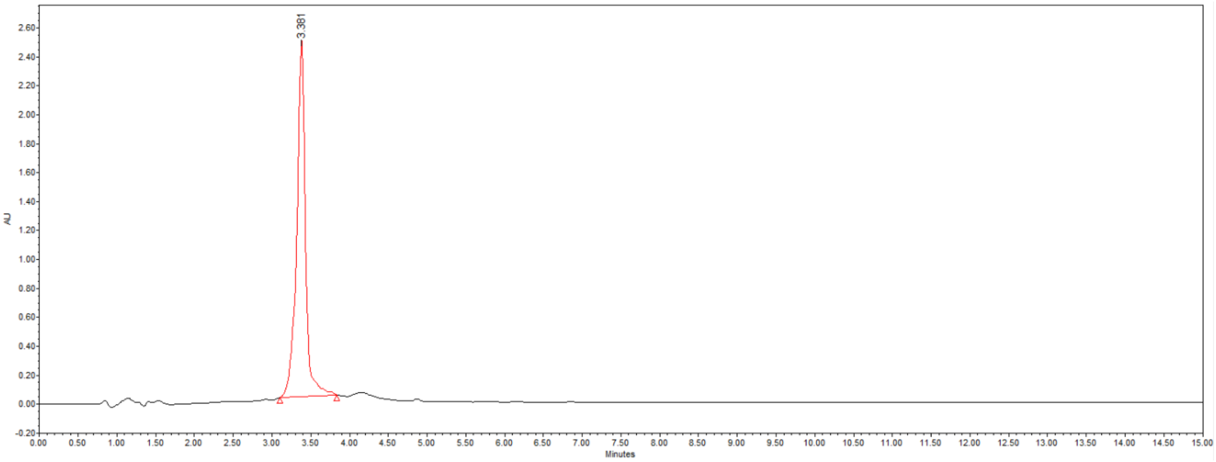


Figure S 13. RP-HPLC spectrum of compound **17**

| Target | Mass Found | Error PPM | Compound |
|---|---|---|---|
| 1952.8824 | 1952.88 | 3.4 | Found |

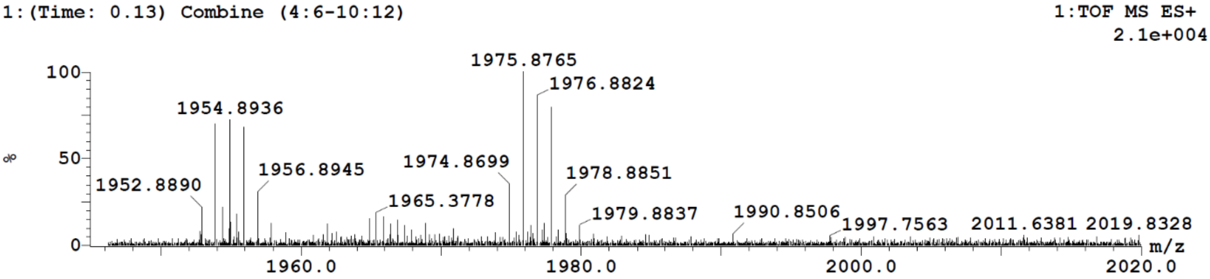


| Mass | PPM | i-FIT (norm) | Formula | Calc. Mass |
|---|---|---|---|---|
| 1952.8890 | 3.4 | 0.0 | C79 H130 N19 O38 | 1952.8824 |

Figure S 14. HRMS spectrum of compound **17**

## Compound 18

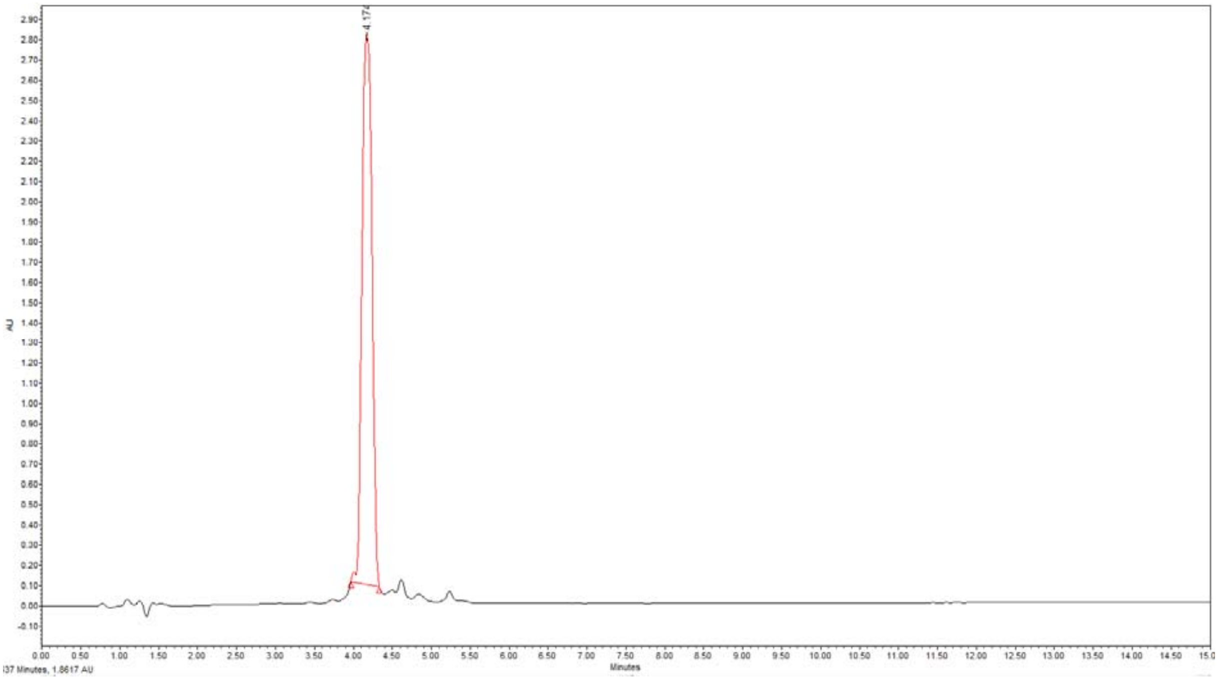


Figure S 15. RP-HPLC spectrum of compound **18**

| Target | Mass Found | Error PPM | Compound |
|---|---|---|---|
| 1996.9464 | 1996.95 | -1.8 | Found |

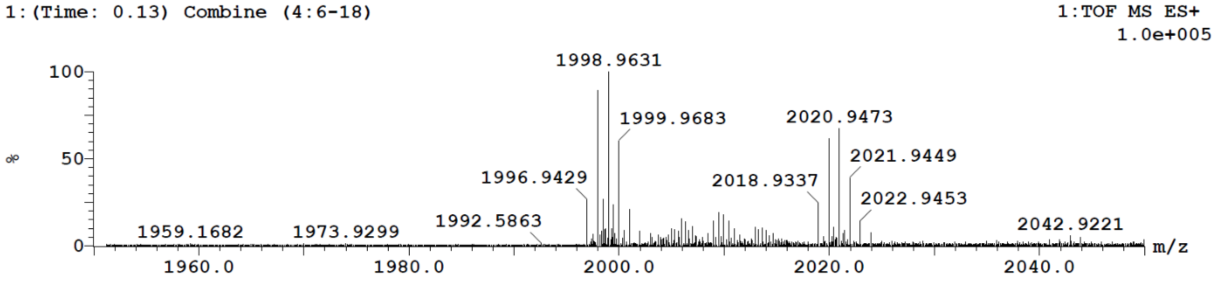


| Mass | PPM | i-FIT (norm) | Formula | Calc. Mass |
|---|---|---|---|---|
| 1996.9429 | -1.8 | 1.5 | C83 H134 N23 O34 | 1996.9464 |

Figure S 16. HRMS spectrum of compound **18**

Compound **19**

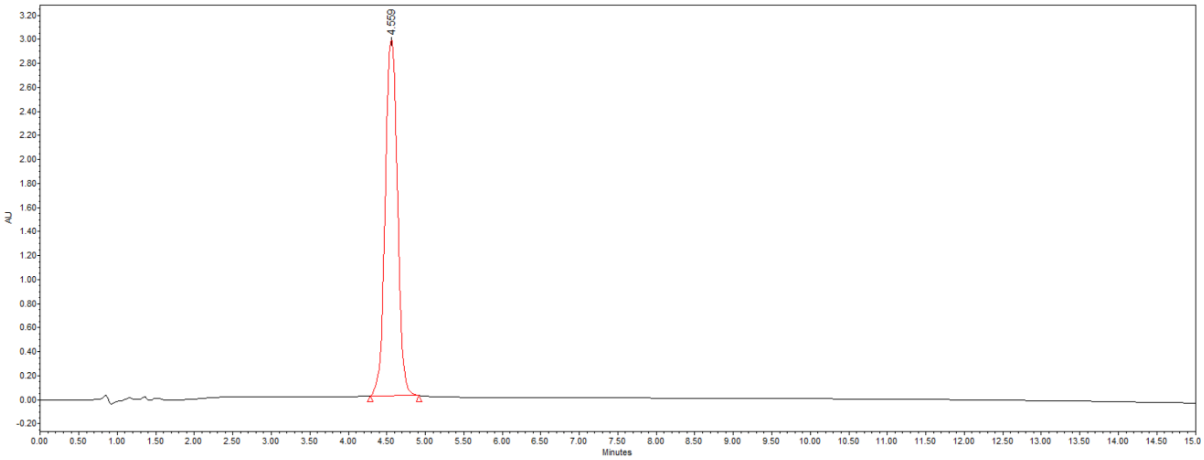


Figure S 17. RP-HPLC spectrum of compound **19**

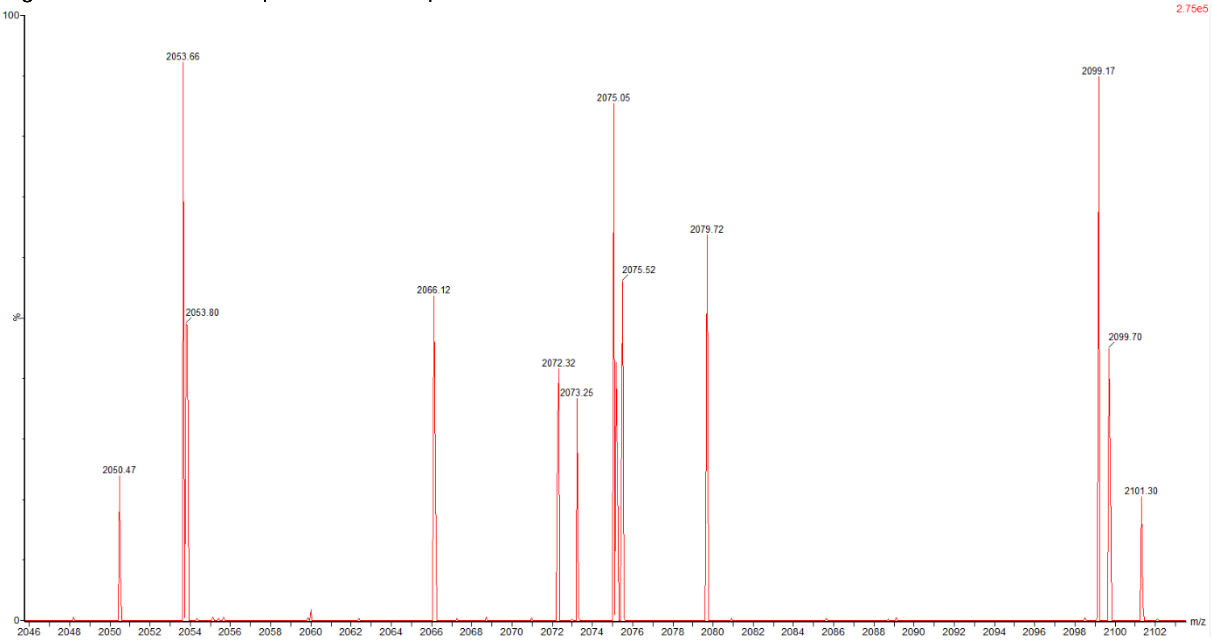


Figure S 18. ESI-MS spectrum of compound **19**

## Compound **20**

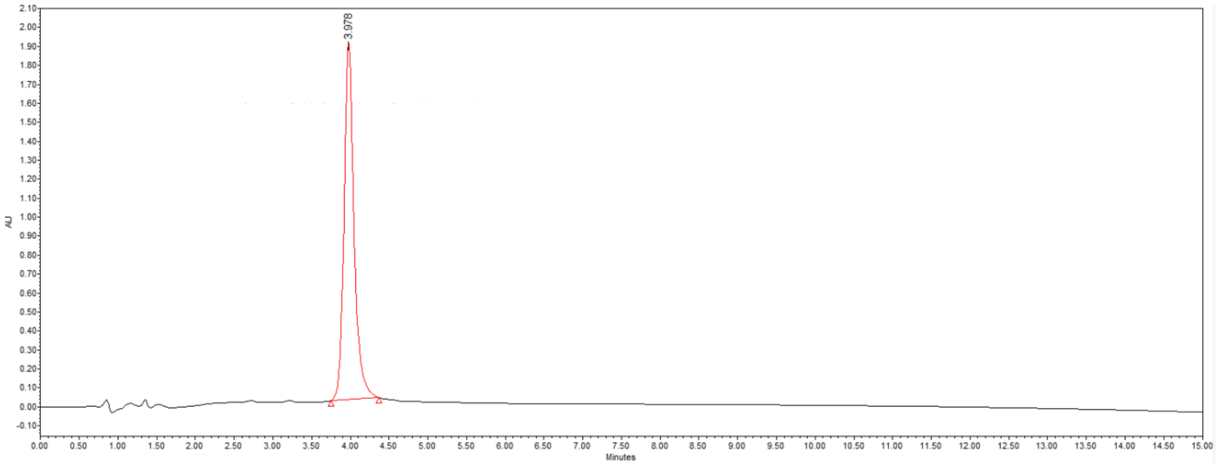


Figure S 19. RP-HPLC spectrum of compound **20**

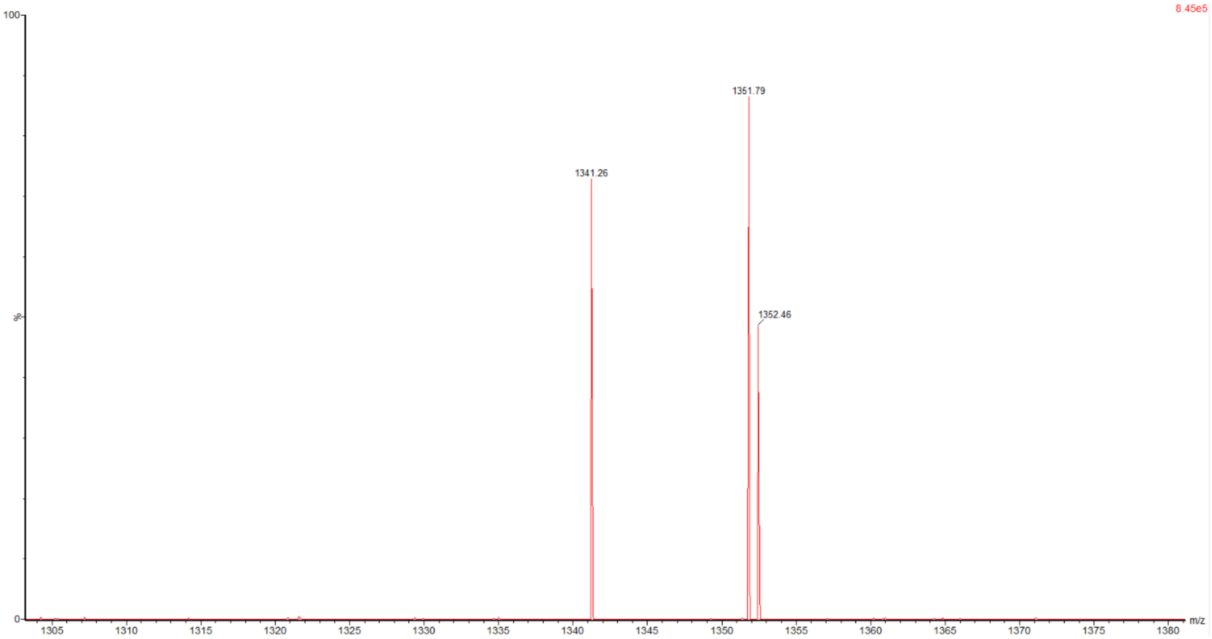


Figure S 20. ESI-MS spectrum of compound **20**

## Isothermal Titration Calorimetry: Titration curves and thermograms

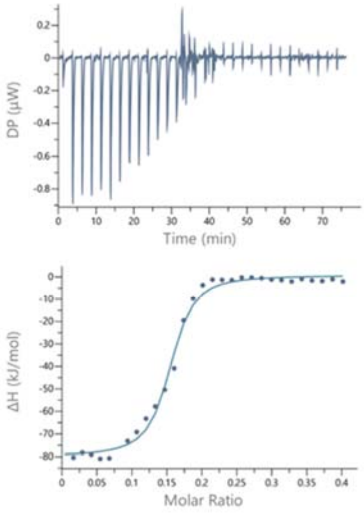


Representative thermogram (Microcal Peak-ITC) obtained by injections of glycocluster **15** at 140 μM in a solution of LecB (70 μM).

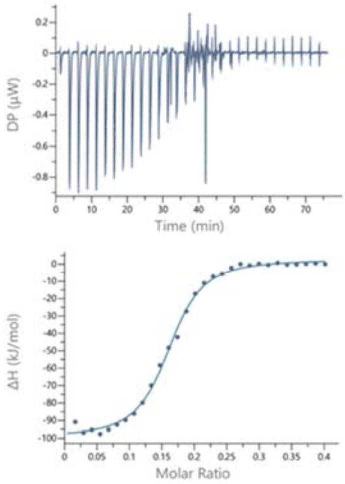


Representative thermogram (Microcal Peak-ITC) obtained by injections of glycocluster **16** at 140 μM in a solution of LecB (70 μM)

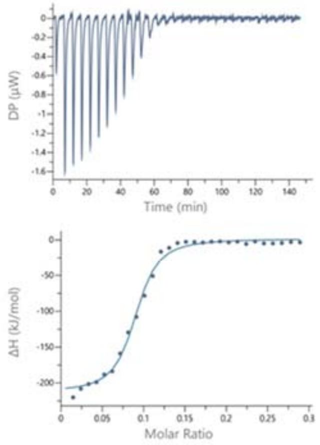


Representative thermogram (Microcal VP-ITC) obtained by injections of glycocluster **19** at 20 μM in a solution of LecB (31 μM)

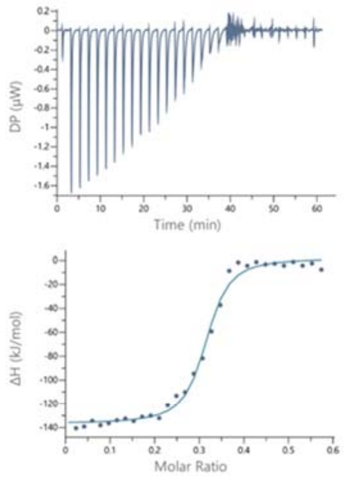


Representative thermogram (Microcal Peak-ITC) obtained by injections of glycocluster **17** at 100 μM in a solution of LecA (35 μM)

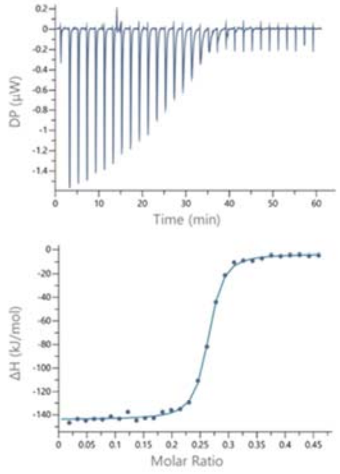


Representative thermogram (Microcal Peak-ITC) obtained by injections of glycocluster **18** at 80 μM in a solution of LecA (35 μM)

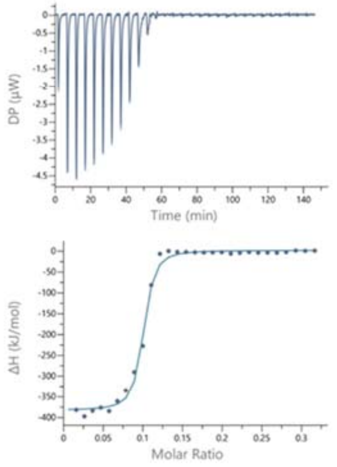


Representative thermogram (Microcal VP-ITC) obtained by injections of glycocluster **20** at 45 μM in a solution of LecA (31 μM)

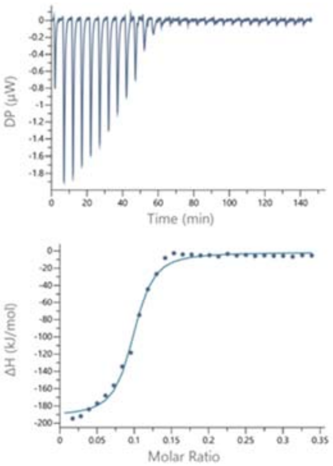


Representative thermogram (Microcal VP-ITC) obtained by injections of glycocluster **11** at 50 μM in a solution of LecB (32 μM)

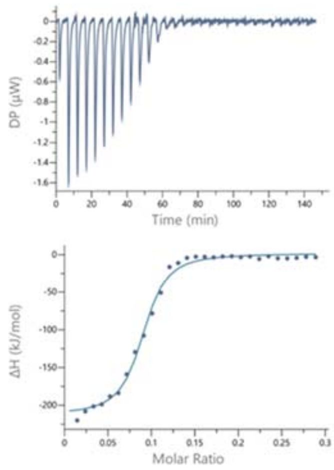


Representative thermogram (Microcal VP-ITC) obtained by injections of glycocluster **12** at 40 μM in a solution of LecB (30 μM)

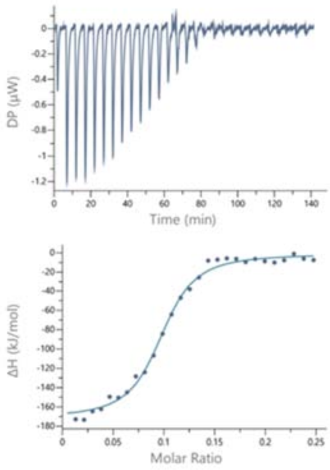


Representative thermogram (Microcal VP-ITC) obtained by injections of glycocluster **13** at 40 μM in a solution of LecB (34 μM)

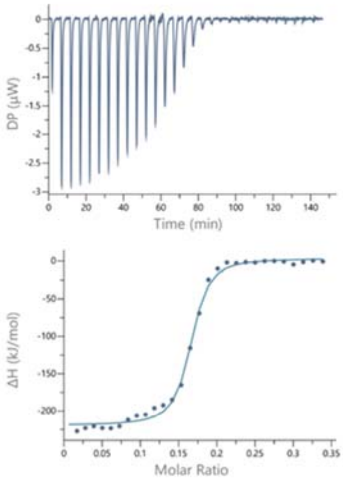


Representative thermogram (Microcal VP-ITC) obtained by injections of glycocluster **11** at 50 μM in a solution of LecA (32 μM)

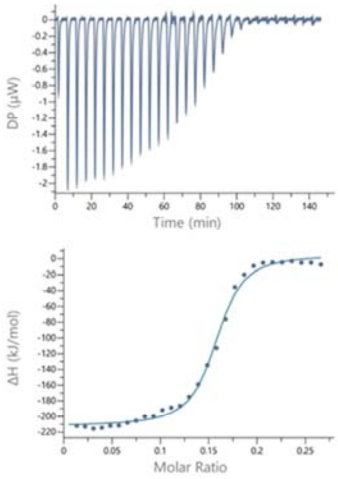


Representative thermogram (Microcal VP-ITC) obtained by injections of glycocluster **13** at 40 μM in a solution of LecA (33 μM)

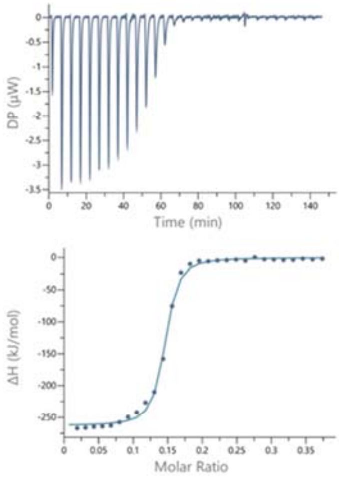


Representative thermogram (Microcal VP-ITC) obtained by injections of glycocluster **14** at 50 μM in a solution of LecA (29 μM)

## References


(1) Singh, Y.; Renaudet, O.; Defrancq, E.; Dumy, P. *Org. Lett.* **2005**, *7*, 1359–1362.

(2) Ribeiro, P.; Villringer, S.; Goyard, D.; Coche-guerente, L.; Höferlin, M.; Renaudet, O.; Römer, W.; Imberty, A. *Chem. Sci.* **2018**, *9*, 7634–7641.

(3) Berthet, N.; Thomas, B.; Bossu, I.; Dufour, E.; Gillon, E.; Garcia, J.; Spinelli, N.; Imberty, A.; Dumy, P.; Renaudet, O. *Bioconjug. Chem.* **2013**, *24*, 1598–1611.
